# The Deafness of Pregnancy and the Post Parturient State

**Published:** 1884-06

**Authors:** Frank Dudley Beane

**Affiliations:** New York City


### Clinical Reports.

### The Deafness of Pregnancy and the Post Parturient State —An Unique History.

By Frank Dudley Beane. A. M., M. D., of New York City.

The literature of the deafness of pregnancy is not so replete with clinical records that the details of the following cases, which have fallen under my observation, will be likely to fail of interest.

*Case* 1.—Mrs. B., æt. 71 years, U. S., widow, the mother of thirteen children, had enjoyed perfect hearing power till after the birth of her seventh child, about forty-two years ago. Each labor had been normal and comparatively easy, and the lyings-in free from complications. After the above-mentioned birth, slowly progressive deafness made its appearance, unattended by pain, discharge or throat affection. After a short time tinnitus was added to the deafness, and became very pronounced. No constitutional or other known cause for the deafness, which has been profound for many years.

PRESENT CONDITION.

*Aerial Conduction.*—Acoumeter and tuning-fork$=\frac{0}{15}$m. Voice, shouting in ears.

*Bone Conduction.*—Greatly diminished.

*Membran. Tympan.*—Both normal in color, lustre, "spot," position and movement, considering patient's age; slight congestion along mallei.

*Eustachian Tubes.*—Patent.

*Naso-Pharynx.*—Normal.

*Case* 2.—Mrs. E. W., æt. 52 years, U. S., widow, daughter of the preceding, never had otorrhœa, injury, or aural trouble till after marriage. Just after the birth of her second child, being then in her twenty-first year, deafness gradually appeared and slowly increased during a period extending over four or five years, and has remained stationary since, with, possibly, a very slight increase. Constant and marked tinnitus.

PRESENT CONDITION.

*Aerial Conduction.*—R. and L. Acoumeter, $\frac{0}{15}$m. Voice (loud), $\frac{1}{15}$m. Whisper, $\frac{0}{15}$m. Tuning-fork, o.

*Bone Conduction.*—Acoumeter and fork, barely perceptible.

*Membran. Tympan.*—Both are normal in color, lustre (for her age), position and movement; malleus distinct and movable (Siegle) on either side.

*Eustachian Tubes.*—Unobstructed.

*Naso-Pharynx.*—Normal.

Her labors (in all eleven) were free from complications. No constitutional disease; no illness of any moment. Deafness not due to "colds" or "earaches."

*Case* 3.—Mrs. C. A., æt. 50 years, U. S., widow, sister to the preceding. No injury, otorrhœa or aural disease till after the birth of her first child, she then being twenty years old, when slight deafness in both ears was noticed. After each succeeding birth (seven in all) the deafness increased, till, after the last, it reached its maximum, its present degree. It has remained unchanged for the past sixteen years at least. Labors easy, and no sequelæ. Had occasional "colds in the head" but no "earaches." Tinnitus has been a prominent and most annoying symptom, as in the preceding cases.

PRESENT CONDITION.

*Aerial Conduction.*—Both ears: Acoumeter and whisper $= \frac{0}{15}$m. Voice (loud) $\frac{1}{15}$m. Tuning-fork$=$o.

*Bone Conduction.*—Very greatly diminished.

*Membran. Tympan.*—Both dull and non-transparent; considerable congestion at periphery and along the head and handle of the mallei; slight "spot" just below and in front of umbo on the left side; none on the right; free movement of the membrane and mallei on using Siegle's instrument.

*Eustachian Tubes.*—Obstructed, but rendered pervious by strong politzeration.

*Naso-Pharynx.*—Follicular hypertrophy and general thickening of the pharyngeal mucosa, but no catarrh.

The history of deafness in this family extends to one son (and brother) who is exceedingly deaf, the result of a chronic suppurative otitis media.

REMARKS.

Perhaps the most striking feature is the occurrence of this form of deafness in three members of a family and in regular descent. In each case there is marked involvement of the nerve of audition, though, strictly speaking, these cannot be called cases of "nervous deafness" pure and simple. Cases 1 and 2 more closely approach such a form, while Case 3 possesses a decided element of catarrh of the middle ear. Possibly if we could carefully examine the tympanum in the other cases, we might discover a partial anchylosis of the stapes to the margin of the fenestra ovalis, which would largely account for the symptoms. Such a discovery would then place all the cases in the category of otitis media catarrhalis sicca. Aural pathology is ever advancing; and, perhaps, it behooves us not too hastily to attribute to lesion of the labyrinth (in some of its phases) cases of deafness with apparent normal conducting apparatus. Drs Sexton and Burnett very carefully emphasized this point in their late estimate of the value of the tuning-fork in aural diagnosis.*

But little has been written upon the subject here under consideration. Many works upon otology refer to shock, sudden

* Paper and discussion before the Amer. Otol. Soc., July 17, 1883.

and large hemorrhages, severe mental emotion, pronounced general and nervous exhaustion, general and marked anæmia as causes of nervous deafness.

One of, if not the first, authorities to draw attention to the deafness of the post parturient state was William Wilde,† who said: "Many females have become deaf immediately after parturition. In such cases I have generally observed a speckled opacity of the membrane."

The next reference to the subject came from the pen of the learned Toynbee.‡

Though several times quoted, it is so pregnant with interest that it may not be amiss to incorporate it here:

"Mrs. B., aged 40, pale, and of a nervous temperament, consulted me in 1850 on account of complete deafness in both ears. She stated that she had married in India ten years previously, and at the time of her marriage she could hear perfectly well. On the occasion of her first confinement, previous to which her hearing was still perfect, she suffered a good deal from exhaustion, and this was followed by a great degree of deafness, so that she could scarcely hear what was said to her, even when the voice was raised. Upon getting up, and growing much stronger, the deafness was so much relieved that she merely required to be spoken to a little louder than usual. During each successive confinement in India, amounting in all to four, the deafness greatly increased, and after each recovery became more permanent, until, on the last occasion, she remained as deaf as at present, when she is obliged to have recourse to signs. Indeed, she has never heard the voice of her younger children, and can only by the movements of their lips understand their words."

Dr. Roosa § says: "Just how it is that pregnant women are so often affected by a proliferous inflammation of the middle ear, I am unable to say; but it is a fact, that many women have told

† Pract. Obser. on Aural Surgery, Phila., 1853, p. 275.
‡ The Diseases of the Ear: Their Nature, Diagnosis and Treatment; London, 1860; p. 370.
§ A Practical Treatise on Diseases of the Ear; New York, 1881, p. 286.

me that they traced their impairment of hearing to their first pregnancy, and that they became worse at the birth of each child."

Dr. W. W. Moreland, of Boston, has described two interesting cases in the Trans. Amer. Otol. Soc. for 1869, pp. 17, et. seq.

In the same "Transactions," for 1878, p. 180, Dr. Samuel Sexton reported: "Deafness during pregnancy, Dr. Pierce, of Manchester, thinks begins with that condition. Mr. Lennox Brown regards such cases as common, and believes they are not catarrhal, but a thickening of the mucous membrane. Sometimes they are nervous in character."

Buck* says: "In a few cases the loss of power of hearing takes place in a gradual manner, and without any attendant symptoms that might throw light on the cause of such loss. * * * * In this category may be placed those cases of total loss of power of hearing, which are observed in women shortly after confinement."

Pomeroy† devotes a little more than one page to the subject, referring to Wilde, Toynbee and Sexton, as above quoted.

Politzer, in his most admirable treatise,‡ refers to the connection existing between aural diseases and affections of the unimpregnated uterus, but makes no mention of the deafness of gestation and parturition. Nor do Bonnafont,§ Von Trolstch,|| Turnbull,¶ Hinton,** Burnett,†† Jones,‡‡ or Miot,§§ devote any space to the affection.

The etiology of this disease is necessarily involved in much darkness, in those cases unattended by signs of middle ear inflammation, where the deafness slowly progresses and no throat

* Diagnosis and Treatment of Ear Diseases; New York, 1880 (Wood's Library) pp. 390–391.
† The Diagnosis and Treatment of Diseases of the Ear; New York, 1883 pp. 371 and 372.
‡ A Text Book of the Diseases of the Ear; Phila., 1833, p. 688.
§ Traite des Maladies de l'Oreille; Paris, 1860.
|| A Treatise on Diseases of the Ear; New York, 1869.
¶ A Clinical Manual of Diseases of the Ear; Phila., 1872.
** The Questions of Aural Surgery; London, 1874.
†† The Ear: Its Anatomy, Physiol. and Diseases; Phila., 1877.
‡‡ Treatise on Aural Surgery; London, Dublin, 1881.
§§ Traite des Malad. de l'Oreille; Paris, 1882.

or nasal complication exists. When it appears suddenly, is complete from the first or very rapidly becomes so, we are justified, in the present state of our knowledge, in considering the internal ear at fault.

Regarding treatment, the advice of Dr. Roosa should make a special impress upon the mind of the general practitioner: "I am now in the habit of warning such patients that great attention should be paid to their throat and ears, by means of gargles and Politzer's method, during the period of utero-gestation."

This treatment should be continued awhile after delivery, till every vestige of impairment of hearing has disappeared. Instead of Politzeration, the Valsavian method may be substituted. Cases which do not promptly improve under this treatment, with a proper care to the maintenance or recovery of general health, may often obtain relief and cure at the hands of the specialist. The prevention of undue hemorrhage at delivery, and measures toward securing a favorable lying-in and prompt recovery, may be important prophylactic factors.

